# Screening of Induced Mutants Led to the Identification of Starch Biosynthetic Genes Associated with Improved Resistant Starch in Wheat

**DOI:** 10.3390/ijms231810741

**Published:** 2022-09-15

**Authors:** Ahsan Irshad, Huijun Guo, Shoaib Ur Rehman, Jiayu Gu, Chaojie Wang, Hongchun Xiong, Yongdun Xie, Shirong Zhao, Luxiang Liu

**Affiliations:** 1 National Engineering Laboratory of Crop Molecular Breeding, National Center of Space Mutagenesis for Crop Improvement, Institute of Crop Sciences, Chinese Academy of Agricultural Sciences, Beijing 100081, China; 2 Institute of Plant Breeding and Biotechnology, Muhammad Nawaz Sharif University of Agriculture, Multan 66000, Pakistan

**Keywords:** expression analysis, mutant population, resistant starch, wheat

## Abstract

Several health benefits are obtained from resistant starch, also known as healthy starch. Enhancing resistant starch with genetic modification has huge commercial importance. The variation of resistant starch content is narrow in wheat, in relation to which limited improvement has been attained. Hence, there is a need to produce a wheat population that has a wide range of variations in resistant starch content. In the present study, stable mutants were screened that showed significant variation in the resistant starch content. A megazyme kit was used for measuring the resistant starch content, digestible starch, and total starch. The analysis of variance showed a significant difference in the mutant population for resistant starch. Furthermore, four diverse mutant lines for resistant starch content were used to study the quantitative expression patterns of 21 starch metabolic pathway genes; and to evaluate the candidate genes for resistant starch biosynthesis. The expression pattern of 21 starch metabolic pathway genes in two diverse mutant lines showed a higher expression of key genes regulating resistant starch biosynthesis (*GBSSI* and their isoforms) in the high resistant starch mutant lines, in comparison to the parent variety (J411). The expression of *SBEs* genes was higher in the low resistant starch mutants. The other three candidate genes showed overexpression (*BMY*, *Pho1*, *Pho2*) and four had reduced (*SSIII*, *SBEI*, *SBEIII*, *ISA3*) expression in high resistant starch mutants. The overexpression of *AMY* and *ISA1* in the high resistant starch mutant line JE0146 may be due to missense mutations in these genes. Similarly, there was a stop_gained mutation for *PHO2*; it also showed overexpression. In addition, the gene expression analysis of 21 starch metabolizing genes in four different mutants (low and high resistant starch mutants) shows that in addition to the important genes, several other genes (phosphorylase, isoamylases) may be involved and contribute to the biosynthesis of resistant starch. There is a need to do further study about these new genes, which are responsible for the fluctuation of resistant starch in the mutants.

## 1. Introduction

Starch is the main part of the wheat grains (70% to 80% of the dry weight of seed) and any change in their nutritional composition can help to provide benefits to a lot of people. The composition of the nutrients in starch depends upon the ratio of amylopectin and amylose [1]. Amylopectin represents 70% to 80% of the starch dry weight in wheat; in addition, it is easily digested by humans and animals. The remaining part of the starch, 20% to 30%, is amylose; and this part helps to form complexes that assist resistance in the digestion of food [2,3].

The portion of the starch that passes through the small intestine in undigested form from the healthy person is called resistant starch [4]. By using the resistant starch, there will be less chance of many diseases, such as obesity, cardiovascular, diabetes, and colon tumors [4,5,6,7]. Many starch synthesis genes are involved in the formation of resistant starch synthesis during grain formation [8]. Resistant starch content increases in rice by a null mutation in the *SSIIa* gene [9]. Similarly, in rice, wheat, and barley, *SBEIII* knocking out or silencing the gene helps to increase resistant starch [10,11,12,13]. The functional loss of the starch synthase gene *GBBSS* decreases the percentage of resistant starch in wheat grains [14]. It is a complex quantitative trait that is controlled by many genes. Seventeen quantitative trait loci (QTLs) have been identified at the different chromosomes of rice for resistant starch [15,16]. In addition, 10 SNPs have been identified at chromosomes 1,3, 6, and 7 that are associated with resistant starch in lentils [17]. Moreover, 40 SNP markers related to resistant starch have been identified through GWAS by screening 209 genotypes of spring barley [18]. Two additive QTLs at chromosome 4A have been identified out of 14 QTLs; this showed variation in the phenotype of wheat [19].

Variations have been produced in many crops through different chemical agents. Ethyl methane sulphonate (EMS) is the main agent for producing variations in different crops [20]. It helps with the mispairing of a guanine base with thiamine instead of cytosine, and causes a transition from G/C to A/T [21]. This biotechnological approach is more preferable than other mutagens as it causes a huge number of mutations by giving multiple alleles of a specific gene in a small population. Many genes of starch biosynthesis produce novel allelic variations by using EMS-induced mutagenesis. By targeting the *GBSSI* loci in wheat, many partial waxy and full waxy phenotypes have been produced [22]. Similarly, many other starch metabolic genes, such as *SBEIIa*, *SBEIIb*, and *SSIIa*, have been studied in wheat for the development of high resistant starch and low resistant mutant lines [3,23].

In wheat, seed formation is divided into three stages: (1) the division and expansion stage (0–14 days after anthesis); (2) the grain-filling stage (14–24 days after anthesis); and (3) the maturation stage (28 days after anthesis) [24]. The maximum starch accumulation in the seed is at the grain-filling stage; later, its concentration gradually decreased [18]. The genes involved in sucrose hydrolysis showed maximum expression and significantly increased at the grain-filling stage [25]. Different studies have illustrated that transcription factors regulate the starch-related gene expression at different stages in wheat [25]. During grain development, the *ZIP* TF family is preferentially expressed to regulate starch synthesis [26]. In transgenic wheat, the overexpression of *TaMYB13-1* and *bHLH* helps to increase starch in wheat [26,27]. Different studies have reported the increasing of starch content through chemical, physical, and other genetic regulations [28]. For genome-wide analysis, we used a set of mutants having the same genetic background and a wide range of variations in resistant starch content to understand resistant starch biosynthesis.

**Figure 1:**
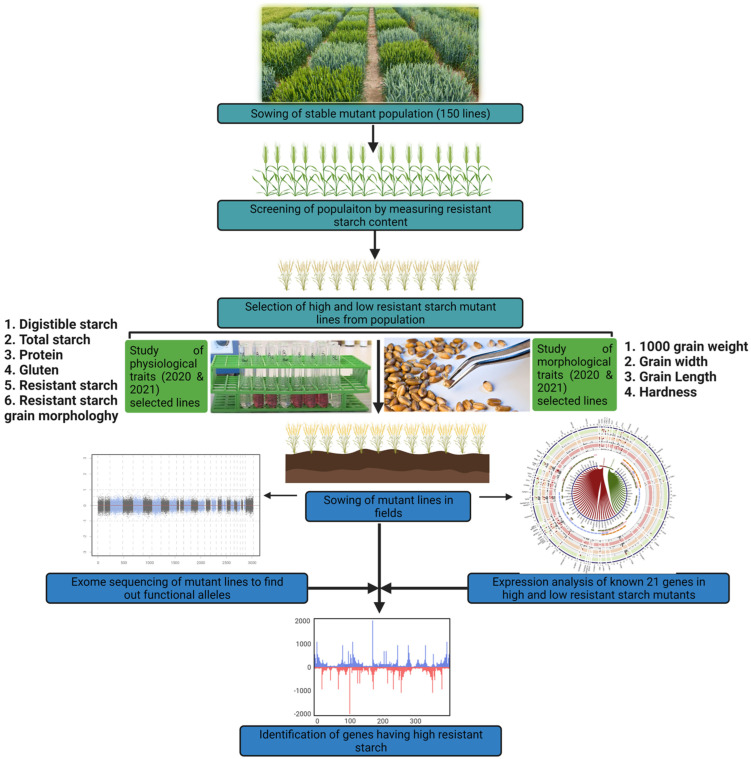
A schematic diagram of the overall workflow.

In the present study, the EMS stable population was screened, whose parent variety was J411; it showed variation for resistant starch content. This stable population consisted of one hundred and fifty mutant lines. Further, two high resistant mutant lines and two low resistant starch mutant lines were used to study quantitative gene expression patterns of 21 starch metabolic pathway genes during seed development. The overall workflow is given in Figure 1.

## 2. Results

### 2.1. Mutant Population Screening for Resistant Starch

The EMS mutant library was screened, which was the uniform population. This mutant population was consisting of 150 mutant lines. These mutant lines were sown in the experimental area of the Institute of Crop Sciences, Beijing with three replications. From these 150 mutant lines, samples of each replication were collected at the maturity stage of the crop. Resistant starch content, digestible starch, and total starch content were measured from these samples by using the Megazyme kit. One-way ANOVA analysis showed significant variation in the resistant starch content of the lines in the three biological replications. Some lines showed resistant starch of more than 17% and low resistant starch in lines of 0.7%; while the resistant starch of J411 was 1.3%. Similarly, the maximum digestible starch in some mutant lines was 76.2%; while the lowest digestible starch in some mutant lines was 27.4%. The overall results of all the mutant lines are given in Supplementary Figure S1.

### 2.2. High and Low Resistant Starch Mutant Lines

**Figure 2:**
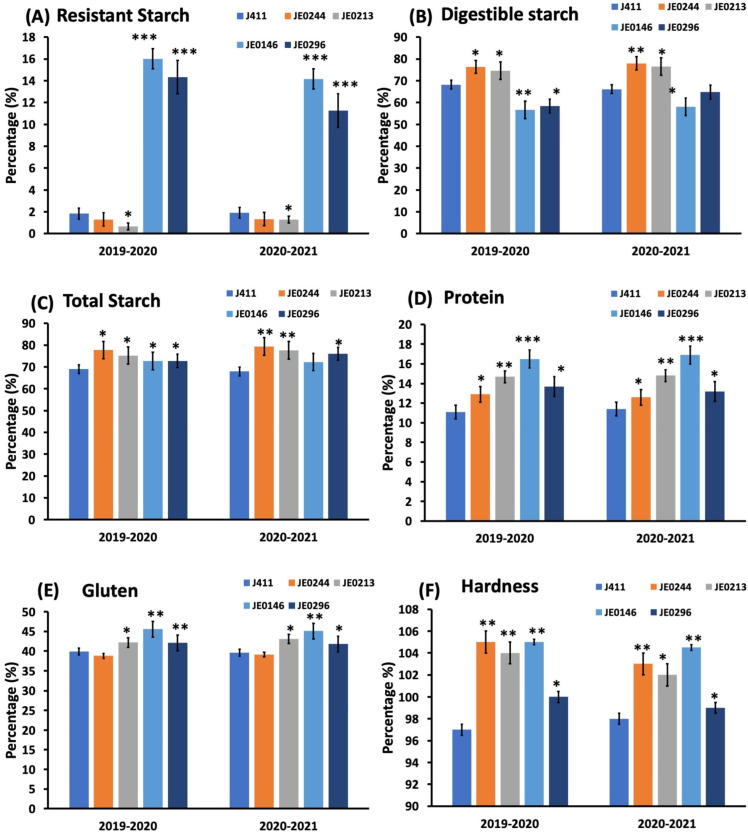
Representative mutants with altered resistant starch content and other physiological traits in the selected lines in 2019–2020 and 2020–2021: (**A**) resistant starch; (**B**) digestible starch; (**C**) total starch; (**D**) protein; (**E**) gluten; and (**F**) hardness. Asterisks (*, **, ***) indicate that the trait mean was significantly different (*p*-value < 0.01) between the WT and mutant lines.

Firstly, two high resistant starch lines (JE0146 and JE0296) and two low resistant starch (JE0244 and JE0213) lines were selected after screening the mutant population. These lines showed a significant difference from WT (J411) whose value was 1.2% in 2019–2020 and 2020–2021 (Figure 2). In both years, the highest resistant starch was in JE0146; its value was 16.5% and 15.3% in 2020 and 2021, respectively. Similarly, in both years, the digestible starch of JE0146 and JE0296 was lower as compared to WT (J411); while digestible starch was higher in low starch mutant lines. All the lines showed a significant difference in total starch as compared to J411. Similarly, for the protein content, gluten, and grain hardness, all the mutants showed a significant difference in both years of data as compared to J411 (Figure 2).

### 2.3. Study of Yield Realted Traits in the High and Low Resistant Starch Mutant Lines

**Figure 3:**
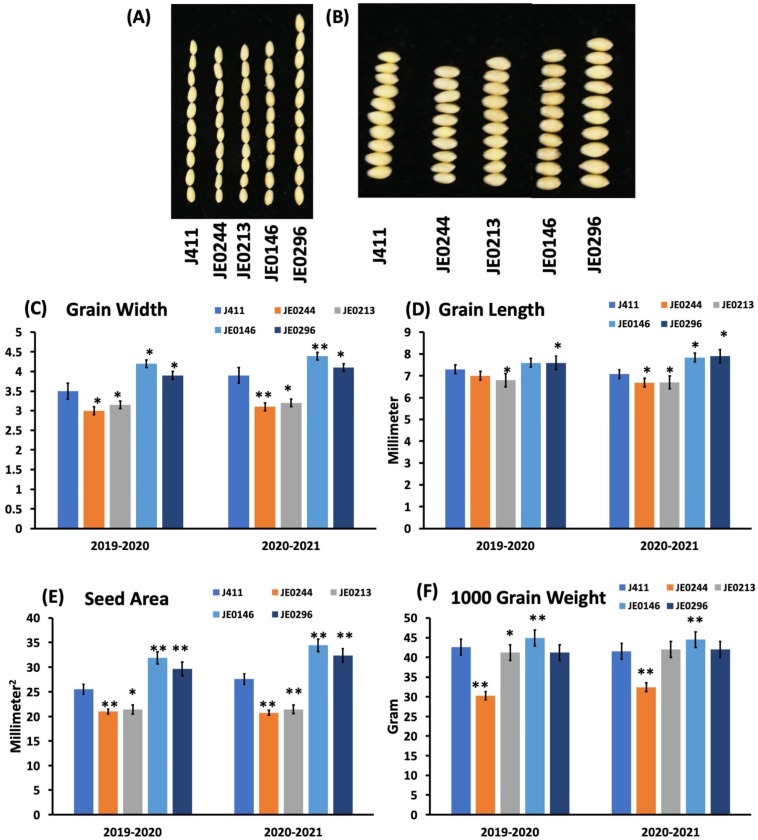
Study of morphological traits in the mutant lines. The graphs show the distribution of morphological traits for the wild-type and resistant starch mutant wheat lines: (**A**) image of the mutant lines and WT for grain length; (**B**) image of the mutant lines and WT for grain width; (**C**) grain width; (**D**) grain length; (**E**) seed area; and (**F**) 1000-grain weight. Asterisks (*, **) indicate that the trait mean was significantly different (*p*-value < 0.01) between the WT and mutant lines.

The images of high starch mutant lines and low starch mutant lines were taken to observe the length and width of the mutant lines (Figure 3). The grain width of the high starch mutant lines was significantly higher than J411; in which, the width of JE0146 was 7.2 mm and 7.6 mm in 2020 and 2021, respectively; while the width of JE0296 was 7.9 mm and 8.1 mm in 2020 and 2021. The grain length of the low resistant starch was lower as compared to J411; while it was higher for the high resistant starch mutant line JE0296, which was significantly higher than J411 (Figure 3). The thousand-grain weight of the high resistant line “JE0146” (43.2 g and 44.6 g in 2020 and 2021, respectively) was significantly higher than J411; while there was no significant difference in both years for JE0296 (Figure 3). Similarly, the 1000-grain weight (TGW) of the low resistant starch mutant lines was lower than J411. It showed a significant difference in both years’ data. In the present study, the lines that had a higher resistant starch content also showed a significant change in TGW; moreover, these lines can be used in the breeding program for the development of high resistant starch varieties (Figure 3). All the mutant lines also showed significant differences in the grain area.

### 2.4. Influences on the Morphology of Starch Granules in High and Low Resistant Starch Mutants

**Figure 4:**
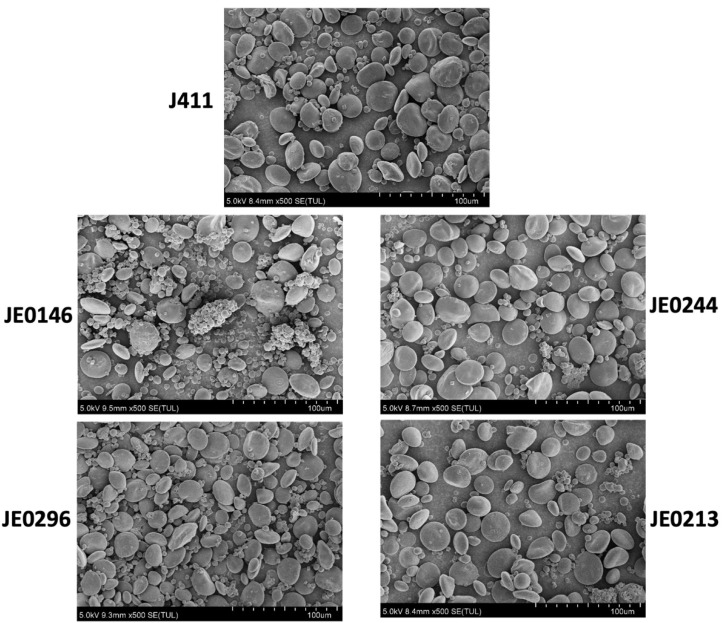
Comparison between the grain morphology of the high and low resistant starch mutants, and the WT (J411). JE0146 and JE0296 are the high resistant starch mutant lines; while JE0244 and JE0213 are the low resistant starch mutant lines.

Pure starch was extracted from wheat flour; a TM4000 scanning electron microscope observed the difference in the morphology of starch granules between the wild-type (J411) and resistant starch mutants (Figure 4). The results showed that compared with the wild type, the starch granules of the mutants were reduced, morphologically shrunk, and decreased in number in the low resistant starch mutant lines (JE0244, JE0213). Similarly, in the high resistant starch mutants (JE0146, JE0296), the size of the granules is bigger than in the WT and there is an increased number of granules. The number of A-granules and B-granules were higher in the high resistant starch mutant lines as compared to J411 (WT).

### 2.5. Expression Analysis for Resitant Starch Mutant Lines

Expression analysis was studied in 21 starch metabolic genes, including the genes responsible for high resistant biosynthesis. Expression analysis was conducted at four seed development stages (6DAA, 12DAA, 18DAA, and 24DAA) between four selected mutants and J411 (WT). These four mutant lines contain about 0.8% and 16.5% resistant starch content. There were fifteen starch biosynthesis genes, in which of the large and small subunits of ADP-glucose pyrophosphorylases (*AGPaseL* and *AGPaseS*): two were granule-bound starch synthase (*GBSSI* and *GBSSII*); four were soluble starch synthase isoforms (*SSI, SSII, SSIII*, and *SSIV*); three were isoforms of branching enzymes (*SBEI*, *SBEII*, and *SBEIII*); and the other four were debranching enzymes with pullulanase (*ISA1*, *ISA2*, *ISA3*, and *PUL*). Further, four starch-degrading genes and two transcription factors (*PHO1*, *PHO2*, *AMY*, *BMY*, *SPA*, and *TaRSR1*) were selected for expression analysis.

### 2.6. High Resistant Starch Mutant Lines Expression Analysis of Starch Metabolic Genes

**Figure 5:**
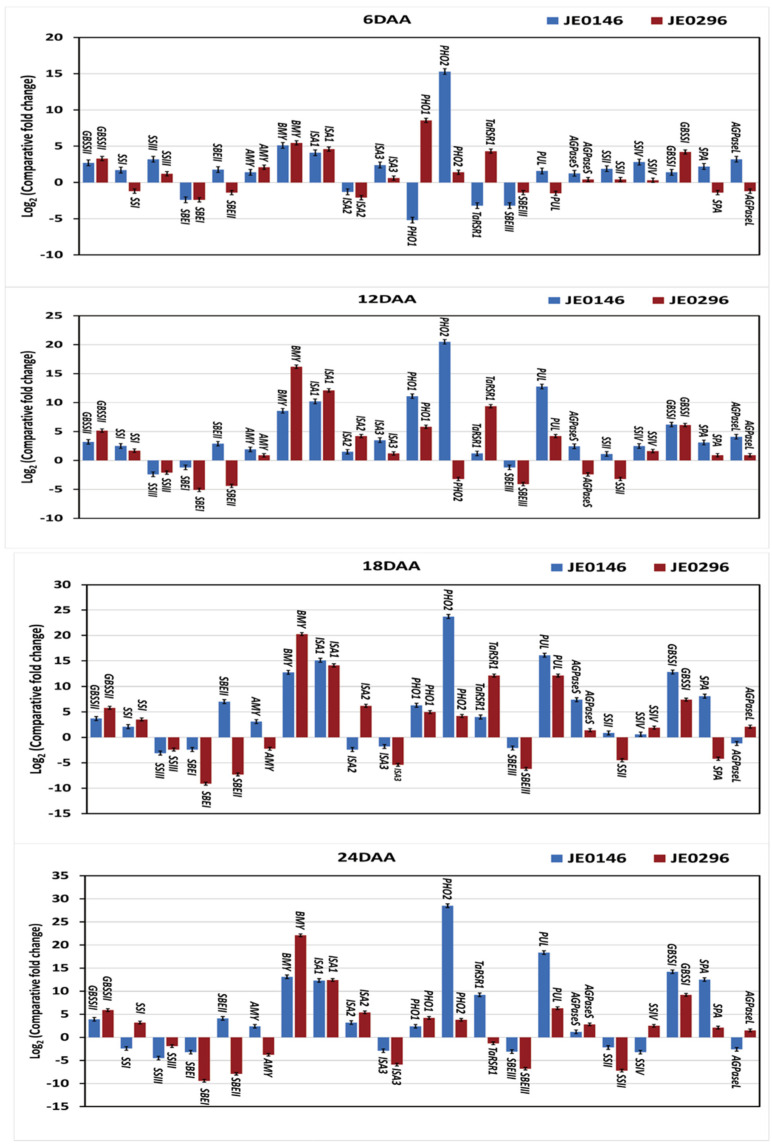
Real-time quantitative expression data (Log2 of fold change) of 21 starch metabolic genes during the seed development in the high resistant starch mutant lines, ‘JE0146 and JE0296’, in comparison to the parent variety, ‘J411’. The seed development stages were 6, 12 18, and 24 days after anthesis (DAA). All the data are represented as mean ± SD from two biological and three technical replicates.

The comparative expression analysis of 21 genes showed that the expressions of twelve genes in JE0146 and nine genes in JE0296 were consistent throughout the seed development stages in the high resistant starch mutant lines with comparison to ‘J411’ (Figure 5). From these genes in both mutant lines, ten genes in JE0146 (*GBSSII, SBEII, AMY, BMY, ISA1, PHO2, PUL, AGPaseS, GBSSI*, and *SPA*) and six genes in JE0296 (*GBSSII, BMY, ISA1, PHO1, SSIV*, and *GBSSI*) showed overexpression; moreover, two genes of JE0146 (*SBEI* and *SBEIII*) and three genes of JE0296 (*SBEI, SBEII*, and *SBEIII*) showed reduced expression during all the seed development stages. The expression of the remaining starch metabolic genes was inconsistent; i.e., either high or low expression during the seed development stages (Figure 5).

### 2.7. Low Resistant Starch Mutant Lines Expression Analysis of Starch Metabolic Genes

**Figure 6:**
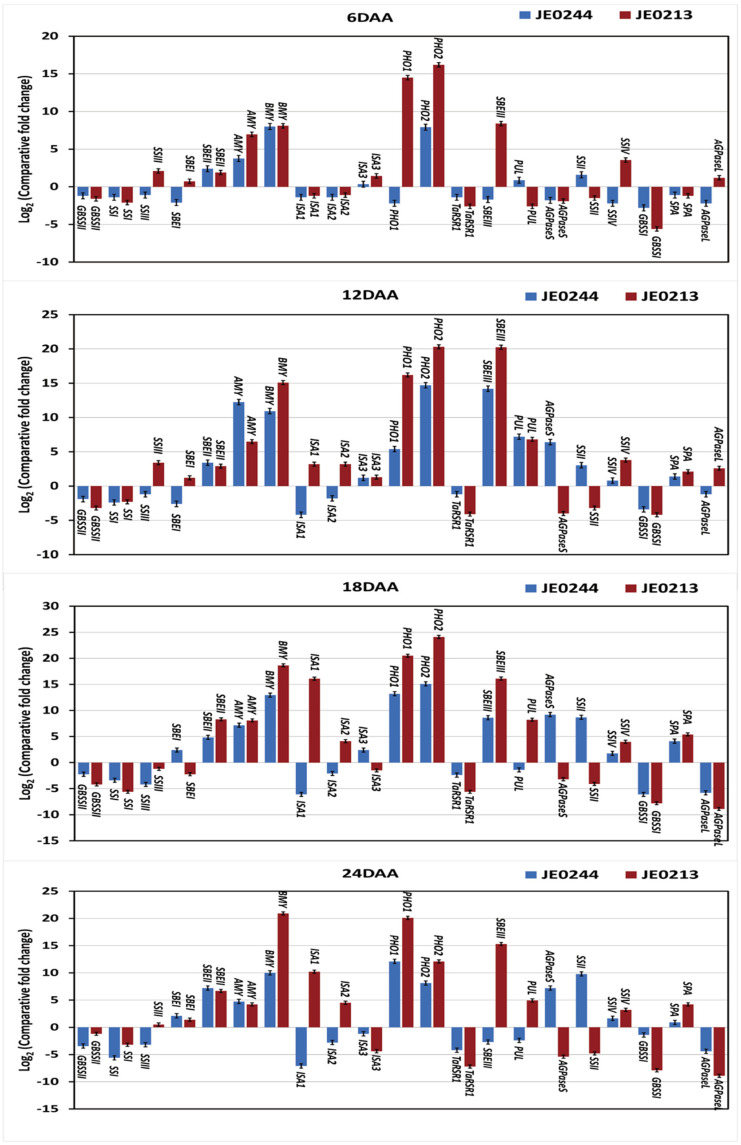
Real-time quantitative expression data (Log2 of fold change) of 19 starch metabolic genes during the seed development in the low starch mutant lines, ‘JE0089 and JE0418’, in comparison to the parent variety, ‘J411’. The seed development stages were 6, 12 18, and 24 days after anthesis (DAA). All the data are represented as mean ± SD from two biological and three technical replicates.

Similarly, a comparative quantitative gene expression analysis of 21 starch metabolic genes identified twelve genes in JE0244 and thirteen genes in JE0213; the expressions of these genes were consistent throughout the seed development stages in the low resistant starch mutant lines in comparison to ‘J411’ (Figure 6). Of the twelve genes of JE0244 and thirteen genes of JE0213, five (*SBEII*, *AMY*, *BMY*, *PHO2*, and *SSII*) and seven (*SBEII*, *AMY*, *BMY*, *PHO1*, *PHO2*, *SBEIII*, and *SSIV*) genes showed overexpression, respectively. In addition, eight genes (*GBSSII*, *SSI*, *SSIII*, *ISA1*, *ISA2*, *TaRSR1*, *GBSSI*, and *AGPaseL*) of JE0244 and six genes (*GBSSII*, *SSI*, *TaRSR1*, *AGPaseS*, *SSII*, and *GBSSI*) of JE0213 showed reduced expression during the seed development stages in the low resistant starch mutant lines (Figure 6). The expression of the remaining starch metabolic genes was not consistent; i.e., either high or low expression during the seed development.

### 2.8. Identification of SNPs and InDels in High and Low Resistant Starch Mutant Lines

**Figure 7:**
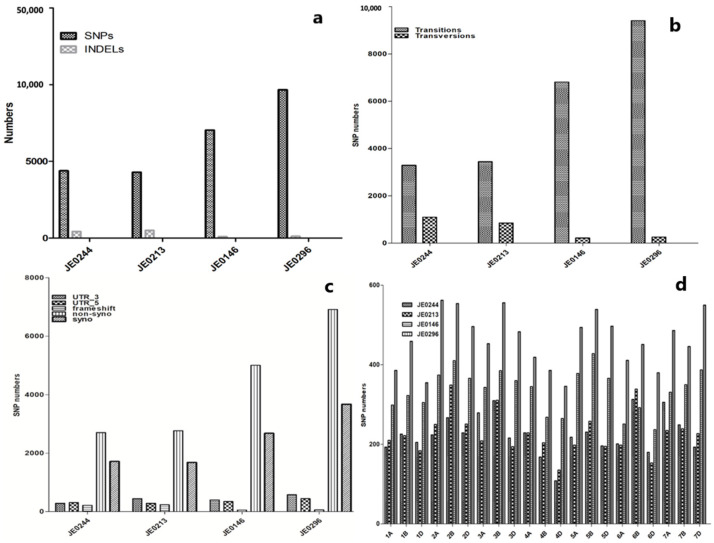
Identification of mutations in the four mutant lines (JE0244, JE0213, JE0146, and JE0296): (**a**) SNPs and Indels; (**b**) transition and transversion mutants in the four mutants; (**c**) Syno, Nsyno, UTR_3, UTR_5, and frameshift mutations; and (**d**) the number of mutations in four mutant lines in the A, B, and D genomes.

Exome sequencing was conducted for the selected high resistant starch mutant lines (JE0146 and JE0296) and low resistant starch mutant lines (JE0244 and JE0213). It was depicted that in the mutant lines, the number of SNPs were more in all four mutant lines than in the Indels. The number of SNPs in JE0296 was higher than the other three mutants. However, the Indels were almost in an equal amount in all the mutant lines. The different types of SNPs between the mutants suggested that there were more transitions than transversions in the four mutants (Figure 7). There were also fewer synonymous mutations in all the mutant lines.

From the four mutant lines, seventeen variants were identified in twenty-one starch biosynthesis genes. Eight variants were discovered in JE0146: five were missense mutations; two were synonymous mutations; and the remaining one was a stop_gained variant. JE0296 had five variants that consist of three synonymous and two missense mutations. Similarly, JE0244 and JE0213 both had two variants (Supplementary Table S1).

The overexpression of *AMY* and *ISA1* in the high resistant starch mutant line JE0146 may be due to missense mutations in these genes. Similarly, there was a stop_gained mutation for *PHO2* and it also showed overexpression. *SBEIII* had a missense mutation with reduced expression. These mutations could be responsible for the high resistant starch in JE0146. JE0296 had two missense mutations in the *AMY* and *SSII* genes; however, their expression was inconsistent. Thus, we can say that there may be some other starch-related genes that may be responsible for high resistant starch content in this line, and that change the expression of other genes. In the low resistant starch mutant line JE0244, there was one missense mutation in *SBE1*, with inconsistent expression; wherein, in the early stages, there was reduced expression and later, overexpression. The overexpression of SBE genes helps to reduce the resistant starch. There was also one conservative_inframe mutation in *SSII* and gene expression increased in the mutant line JE0244. Similarly, there were two mutations in the known 21 genes of JE0213; in which one was a 5_prime_UTR mutation in *SBEI* and due to this, mutation expression was inconsistent. The second mutation was a disruptive_inframe mutation in the *SSII* gene and the expression was reduced.

## 3. Discussion

Wheat is the major staple crop and it is the main source of cereal-based products. The modification of starch composition in wheat by increasing the resistant starch content helps to improve the quality and yield of crops [2]. There has been significant use of chemical mutagenesis for crop functional genomics and breeding research [29]. Artificial mutagenesis technology is an efficient way to improve the crop because of less variation in the natural population for different crops. Therefore, it presents a good opportunity to understand the function of genes in any organism by using the mutants with altered phenotypes [30]. EMS-induced lines have been used to identify a functional mutation for a candidate gene in diploid, tetraploid, and hexaploidy wheat [29,31,32]. The bread wheat variety, J411, used in this study was treated with EMS. In total, 150 uniform mutant lines were used for screening resistant starch content to identify high resistant starch lines and low resistant starch lines.

Different pieces of published literature have shown that the resistant starch contents were in the range from 1% to 18% in different wheat mutants [19]. In this study, some mutant lines showed >16% resistant starch content. Therefore, these mutants will be useful for the study of genome-wide analysis of the genetic and molecular basis for resistant starch variation and help to improve the quality of wheat. The lines which showed high resistant starch had a better weight than the parent variety; in addition, they can be used to improve the resistant starch and yield through breeding techniques in wheat. Different studies showed the relation between resistant starch, total starch, and TGW. Resistant starch enhances slowly in the initial stage of grain development, and the process becomes fast in the medium stage with a total that ceases at the end of the grain-filling stage. The correlation between resistant starch and TGW, and total starch was consistent as shown in previous studies [18]. Granule morphology mainly affects the proportion of amylose and amylopectin concentration. Starch granules are altered in high resistant lines of wheat and maize [18,33]. This altered shape of starch granules is likely to increase the resistant starch proportion in the granules [34].

The expression analysis of 21 starch metabolic genes was conducted by including genes responsible for changing the resistant starch of four altered resistant starch mutants and a parent variety (J411) at four different stages of grain formation (Figure 6 and Figure 7). The overexpression of *GBSSI* in the present study supports the previous results that may be the accumulation of high amylose; this is because *GBSSI* plays an important role by elongating the *a*-amylase chain, which ultimately increases the resistant starch content [35]. Amylose content increased by the overexpression of *GBSSI* in wheat and rice; while the silence of this gene or null mutants produced waxy and partial waxy wheat with a low amount of resistant starch [34,36,37]. Decreasing the activity of SSs, SBEs, and isoamylase helps to increase the content of resistant starch in wheat. In maize, the functional loss of *SSIII* helps to increase the resistant starch content [38]. Similarly, the inhibition of *ISA* significantly increased the amylopectin content in rice [39]. *SBEII* silencing helped to increase the amylose content in wheat, which ultimately increased the resistant starch content [40]. Therefore, this study reveals that the overexpression of the main genes of starch biosynthesis helps to increase the amount of resistant starch.

Amylase (*AMY* and *BMY)* with starch phosphorylases (plastidial; *Pho1* and cytosolic; *Pho2*) plays an important role in starch metabolism with degradation and hydrolysis [41]. These are main starch genes that help in the maintenance of the starch structure and also starch morphology. The alteration of starch was observed in rice and potato by the silencing of starch phosphorylase [42]. Similarly, *AMY* and *BMY* overexpression also altered the starch structure and starch granules. Morphology, with changing the structure of starch granules, affects the baking quality of wheat [43]. *SSI* also plays important role in the increment of resistant starch. In wheat and rice, losing the function of *SSI* helped to increase the resistant starch as well as amylose content [44]. Similarly, the overexpression of *SBEII* decreased the amylose content in potato and increased the amylopectin content [45]. In rice, a negative transcription factor (*RSR1*) was identified [46]. This transcription factor negatively regulates the other starch metabolic genes; consequently, it modulates starch metabolism and starch-associated phenotypes.

## 4. Material and Method

### 4.1. Plant Material

An EMS mutant uniform population was used in the present study, whose parent variety was J411. This uniform population was grown in a field in 2019–2020 at the Institute of Crop Sciences, Chinese Academy of Agricultural Sciences, Beijing. This population consisted of 150 mutant lines. Seed samples were collected from each mutant line and crushed to make the flour for studying the resistant starch content and other traits. Similarly, in 2020–2021, the selected lines were sown and seed samples were collected to study the different physiological and morphological traits.

### 4.2. Measurement of Resistant Starch and Digestible Starch

The resistant starch (RS) and digestible starch (DS) of 150 mutant lines were measured by using a commercial Megazyme kit (K-RSTAR 05/19, Megazyme Int., Wicklow, Ireland) following the manufacturer’s protocol; this being the detailed protocol followed by McClary et al. [47]. Three biologicals with two technical replications were performed for each sample.

Resistant Starch % = ΔA × F × EV/0.1 × 1/1000 × 100/W × 162/180 = ΔA × F × EV/W × 0.90(1)

Digestible Starch % = ΔA × F × EV/0.1 × 1/1000 × 100/W × 162/180
= ΔA × F × EV/W × 0.90(2)

ΔA = absorbance of sample solution read against reagent blank.

F = factor to convert absorbance values to mg glucose.

EV = sample extraction volume (10.3 mL or 100 mL).

0.1 = volume of sample analysed.

1000 = conversion from mg to mg.

100/W = conversion to 100 mg sample.

W = sample weight in mg.

162/180 = factor to convert from free glucose, as determined, to anhydroglucose, as occurs in starch.

### 4.3. Determination of Total Starch

The total starch content was determined using the Megazyme International Starch Assay Kit (K-STAR) as per the manufacturer’s protocol [47]. Three biologicals with two technical replications were performed for each sample with a starch assay kit.

Starch % = ΔA × F × EV/0.1 × D × 1/1000 × 100/W × 162/180
= ΔA × F × EV × D/W × 0.90 (3)

ΔA = absorbance of sample solution read against reagent blank.

F = factor to convert absorbance values to μg glucose (100 μg glucose divided by the GOPOD absorbance value obtained for 100 μg of glucose).

EV = sample extraction volume (10.2 mL for the procedure).

0.1 = volume of sample analysed.

D = further dilution of sample solution.

1/1000 = conversion from μg to mg.

100/W = conversion to 100 mg sample; W: sample weight in mg.

### 4.4. Protein, Gluten Content, and Seed Hardness

A grain analyzer was used to measure the protein, gluten content, and hardness of the seed. A FOSS infratecTM 141 (FOSS Analytical AB, Sweden) grain analyzer was used to measure these traits by using WinISI II v1.50 (InfraSoft International LLC, 2000, Port Matilda, PA, USA) [48]. All these traits were measured in the percentage.

### 4.5. Measurement of Grain Weight, Width, and Length

Samples of seed from each of the mutant lines and WT were collected with three replications for measuring the grain width, grain length, grain area, and thousand-grain weight (TGW). SmartGrain was used for evaluating these traits by following the methodology described in previous studies [49]. TGW was measured in grams, while width and length were measured in millimeters.

### 4.6. Starch Purification and Grain Morphology

Seed starch purification was conducted by following the method described by Hawkin et al. [49]. To study the grain morphology, a Nova NanoSEM 450 (FEI) scanning electron microscope (SEM) was used to watch the A-granules and B-granules.

### 4.7. Exome Sequencing of Mutant Lines

Selected mutants were used to capture the genetic diversity. The four mutant pools were subjected to exon capture and sequencing to compare the polymorphisms between different extreme pools. The process of exon sequencing was followed as described by King et al. [50]. First, the quality of DNA in the mixed pool was detected, and the DNA library was constructed after being qualified; then, the sample library was amplified by PCR; the captured DNA fragments were subsequently eluted and recovered; and finally, the captured DNA fragments were enriched. The abundance was subsequently detected by qPCR; after quality control, high-throughput sequencing was performed on the Illumina platform. Further analyses were followed, as described by Du et al. [51].

### 4.8. Quantitative Gene Expression Analysis

Twenty-one starch metabolic genes were used to perform quantitative expression analysis at four seed development stages (6DAA, 12DAA, 18DAA, and 24DAA) in two low resistant mutant lines (JE0244 and JE0213) and two high resistant mutant lines (JE0146 and JE0296), and the parent wheat variety ‘J411’. The samples were collected from the selected line at 6, 12, 18, and 24 days after anthesis and stored quickly in liquid nitrogen; they were then stored at -80 degrees centigrade for the extraction of RNA. The detailed protocol for RNA extraction and cDNA formation used in this study followed that of Singh et al. [52]. Primer information on these 21 genes was retrieved from Singh et al. [53]. Three technical replicates with two biological replicates were used for quantitative expression analysis in all the stages by using a fast real-time PCR system (Applied Biosystems, Forster City, CA, USA); in addition, *Actin* (GenBank accession no: AAW78915), a housekeeping gene, was used as an internal control. We followed the method of Singh et al. [53]. The ΔΔCT method was used to analyze the relative expression level. The formula used for quantitative expression analysis is given below:

ΔΔCT = ΔCT (a target sample) − ΔCT (a reference sample) = (CT_D_ − CT_B_) − (CT_C_ − CT_A_).

### 4.9. Statistical Analysis

Microsoft Excel formulas were used to calculate the mean value and standard deviation. In order to study the variations in resistant starch and other traits, a one-way analysis of variance (ANOVA) was used. To find the significant difference between the mutant lines and parent variety for all the traits, the Dunnet test was used. For the purpose of these analyses, three biological replications were used for studying all of these traits.

## 5. Conclusions

In this study, 150 mutant lines were used that showed variations in resistant starch content; this useful germplasm can be used for a genome-wide study and help to improve the starch-based nutritional quality in wheat. This study indicates that low resistant starch accumulation in mutant lines may have resulted from the reduced expression of key genes for resistant starch and starch biosynthesis. The differential gene expression analysis in the low and high resistant starch mutant lines in comparison to the parent variety supports the involvement of other starch metabolic pathway genes; these include phosphorylases, isoamylases, etc. in resistant starch biosynthesis. There is a need to find out about starch metabolic genes through the backcrossing of these mutant lines and a developed NILs population for mapping.

## Data Availability

Not applicable.

## Supplementary Materials

The following supporting information can be downloaded at: https://www.mdpi.com/article/10.3390/ijms231810741/s1.
