# Supplementary material for: Screening of Induced Mutants Led to the Identification of Starch Biosynthetic Genes Associated with Improved Resistant Starch in Wheat
Source: Int J Mol Sci. 2022 Sep 15;23(18):10741. doi: 10.3390/ijms231810741 (PMC9502818; doi:10.3390/ijms231810741)
Supplement: Supplementary file 1 [file ijms-23-10741-s001.zip › ijms-1904245-supplementary.pdf]

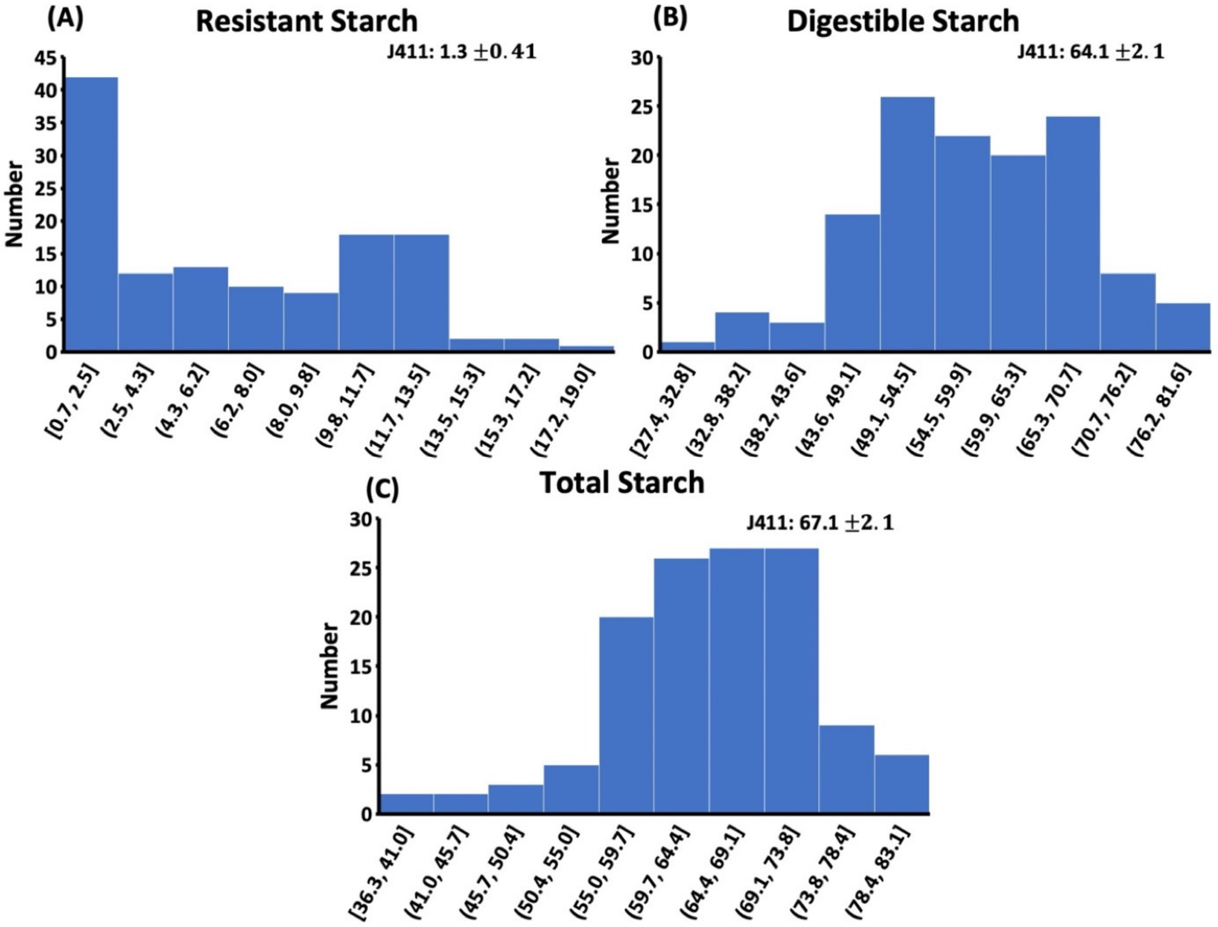

Figure S1. Phenotype distribution of 150 mutant lines.
Variations in Resistant starch content (**A**), Digestible starch (**B**), and total starch (**C**) (among the offspring of 150 mutant lines were displayed. J411 is the parent variety

Table S1. Deleterious mutations detection in the starch biosynthesis genes of high and low starch mutant lines.

No.	Mutant lines	Gene name	Gene ID	Mutation type	position	Nucleotide change	Chromosome
1	**JE0146**	*SSI*	TraesCS6B03G0953800	Synonymous	4041	G/A	6B
		SBEI	TraesCS7A03G1335100	Missense	3041	G/A	7A
		AMY	TraesCS5D03G1053700	Missense	1038	C/T	5D
		ISA	TraesCS7D03G0559000	Missense	639	C/T	7D
		PHO	TraesCS5D03G0900800	stop_gained	3923	C/T	5D
		TaRSR1	TraesCS1B03G0192900	Missense	2293	C/T	1B
		SBEIII	TraesCS7B03G0684800	Missense	17555	C/T	7B
		SSIV	TraesCS1A03G0866200	Synonymous	3446	G/A	1A
2	**JE0296**	*SBEII*	TraesCS2D03G0674400	Synonymous	10504	G/A	2D
		AMY	TraesCS5D03G1053700	Missense	1113	C/T	5D
		SBEIII	TraesCS7A03G0826800	Synonymous	2340	C/T	7A
		SSII	TraesCS7A03G0440500	Synonymous	2947	G/A	7A
		SSII	TraesCS6D03G0680800	Missense	401	C/T	6D
3	**JE0244**	*SBEI*	TraesCS7A03G1335100	Missense	4657	C/T	7A
		SSII	TraesCS1A03G0361500	conservative_inframe	206	T/TCCCCCC	1A
4	**JE0213**	*SBEI*	TraesCS7B03G1268900	5_prime_UTR	32	GCTCCTCCTCCGGCC/G	7B
		SSII	TraesCS1A03G0361500	disruptive_inframe	207	C/CCCCCTA	1A
